# A Large-Scale Study of Fingerprint Matching Systems for Sensor Interoperability Problem

**DOI:** 10.3390/s18041008

**Published:** 2018-03-28

**Authors:** Helala AlShehri, Muhammad Hussain, Hatim AboAlSamh, Mansour AlZuair

**Affiliations:** College of Computer and Information Sciences, King Saud University, Riyadh 11543, Saudi Arabia; hatim@ksu.edu.sa (H.A.); zuair@ksu.edu.sa (M.A.)

**Keywords:** biometrics, fingerprint sensor interoperability, cross-sensor fingerprint matching, fingerprint enhancement

## Abstract

The fingerprint is a commonly used biometric modality that is widely employed for authentication by law enforcement agencies and commercial applications. The designs of existing fingerprint matching methods are based on the hypothesis that the same sensor is used to capture fingerprints during enrollment and verification. Advances in fingerprint sensor technology have raised the question about the usability of current methods when different sensors are employed for enrollment and verification; this is a fingerprint sensor interoperability problem. To provide insight into this problem and assess the status of state-of-the-art matching methods to tackle this problem, we first analyze the characteristics of fingerprints captured with different sensors, which makes cross-sensor matching a challenging problem. We demonstrate the importance of fingerprint enhancement methods for cross-sensor matching. Finally, we conduct a comparative study of state-of-the-art fingerprint recognition methods and provide insight into their abilities to address this problem. We performed experiments using a public database (FingerPass) that contains nine datasets captured with different sensors. We analyzed the effects of different sensors and found that cross-sensor matching performance deteriorates when different sensors are used for enrollment and verification. In view of our analysis, we propose future research directions for this problem.

## 1. Introduction

The use of fingerprints is the oldest and most prevalent method for person identification and authentication. Fingerprint matching problems have been widely explored, and there is significant discussion of fingerprint matching methods in the literature. However, most existing algorithms are designed to work with a specific type of sensor, i.e., the sensor used for enrollment and verification. The rapid growth of new applications and advances in fingerprint sensor technology have given rise to fingerprint sensor interoperability or cross-sensor matching problems, i.e., problems matching an individual’s fingerprints obtained from different sensors.

Fingerprint sensing technologies are based on diverse operational principles such as ultrasound, optical, and capacitive technologies. In ultrasound sensors, the image is based on the response of the acoustic wave bounced off the fingertip. In optical sensors, the finger is placed on the transparent prism surface, and light is reflected from the valleys and absorbed at the ridges. The ridges look dark and the valleys appear bright. The capacitive sensor is composed of small capacitive plates located under the sensor, and air works as the dielectric medium. The strength of the electric field is a function of the distance of the valleys and ridges from the plates [1]. The underlying principles of these technologies present their own form of distortions and inconsistencies, which introduce variations in descriptive features employed by matchers for fingerprint matching and make sensor interoperability a challenge.

Some research has been conducted to show the importance of exploring the impact of changing fingerprint sensors on fingerprint matching systems [2,3,4]. Shimon et al. [2] conducted an empirical study to examine the effect of sensor interoperability on the performance of VeriFinger, a minutiae-based matcher, using a local database (not available publicly) in terms of the false non-match rate (FNMR). Lugini et al. [3] and Mason et al. [4] performed empirical studies using the same local database (not available publicly) captured with four different optical sensors, which have the same resolution. These studies are limited in the sense that Shimon et al. [2] focused only on one minutiae based matcher (VeriFinger), whereas Lugini et al. [3] and Mason et al. [4] employed an interoperable dataset captured with four sensors of the same technology type, which cannot be generalized to sensors of other technology types. Moreover, these studies were conducted on local databases; for new solutions to the problem, it is difficult to reproduce the results obtained in these evaluations and compare the performance of the new algorithms. A study is necessary to answer certain questions, some of which include the following: Which type of features can be robust against different structural and distortion inconsistencies that occur in fingerprints captured with sensors of different technology types and capture types? What is the impact of interoperability on the performance of enhancement algorithms? How does sensor interoperability affect the performance of state-of-the-art fingerprint matching methods? These questions motivated us to analyze the structural inconsistencies of fingerprints captured with different sensors and provide a comparative analysis of state-of-the-art enhancement methods and matching systems to understand the effect of the fingerprint sensor interoperability problem using a public database. Since the database used in this study is available in the public domain, the results can serve as a reference point for comparing the performance of new algorithms.

This study was conducted to analyze the characteristics of fingerprints and the impact of cross-sensor matching on state-of-the-art fingerprint enhancement and matching methods. The matching methods include Minutiae Cylinder-Code (MCC), Bozoroth3 (NBIS software), and VeriFinger, a commercial SDK. The experiments were performed using the public database FingerPass. Specifically, our contributions are as follows:

- An analysis of the structure of fingerprints, which revealed that fingerprints captured with different sensors vary in small-scale structural inconsistencies such as micro-texture patterns and fine ridge details such as width and pores. Ridge patterns, ridge orientations, and minutiae form the main structural component, which is invariant to the technology types and interaction types of sensors.
- An analysis of inter-ridge spacing, which shows that the spacing between successive ridges varies among the impressions of the same finger captured with different sensors and has a significant impact on cross-sensor matching. This issue must be addressed when designing a cross-sensor matching method.
- An analysis of two state-of-the-art enhancement algorithms, which shows that there is a need to develop new enhancement algorithms for cross-sensor matching that are able to preserve ridge patterns and suppress small-scale structural inconsistencies, extraneous ridges, and minutiae.
- An analysis of the three state-of-the-art matching methods, which reveals that there is a need to develop new algorithms for cross-sensor matching. In general, there is a better level of interoperability between optical sensors than capacitive sensors. New feature extraction techniques must be developed for cross-sensor matching, keeping in view the structural components of fingerprints that are variant or invariant to the technology types and interaction types of sensors.

The remainder of this paper is organized as follows: Section 2 describes the fingerprint sensor interoperability problem and feature extraction. Section 3 presents an overview of databases for the fingerprint sensor interoperability problem. Section 4 provides an overview of the methods proposed for the fingerprint sensor interoperability problem, and Section 5 provides an analysis of two fingerprint enhancement methods for cross-sensor matching. Section 6 discusses the impact of fingerprint scaling on cross-sensor matching, and Section 7 presents an analysis of the three matching methods on cross-sensor matching. The conclusion and future research directions are explored in Section 8.

## 2. Fingerprint Sensor Interoperability Problem and Feature Extraction

Fingerprint sensor interoperability addresses the ability of a fingerprint-matching system to compensate for the variability in the fingerprints of an individual acquired using different sensors. Variations in the fingerprints are introduced because of differences in capturing the technology of sensors, interaction type, sensor resolution, and scanning area. Based on technology type, sensors can be categorized as capacitive, optical, temperature differential, touchless, ultrasound, piezoelectric, or multispectral [5]. Each type produces its own type of distortions. In some capturing systems, the path lengths reflect light that varies across the width and length of a fingertip, which can either cause a trapezoidal distortion or generate defocused areas within the acquired fingerprint. Trapezoidal distortion refers to differences that occur in an image when a part is wider than the rest of the image. Capacitive sensors suffer from noise and grid artifacts and are sensitive to salt from sweat and other contaminants. Consequently, sensors cause different types of distortions because of the differences in technology types.

**Figure 1:**
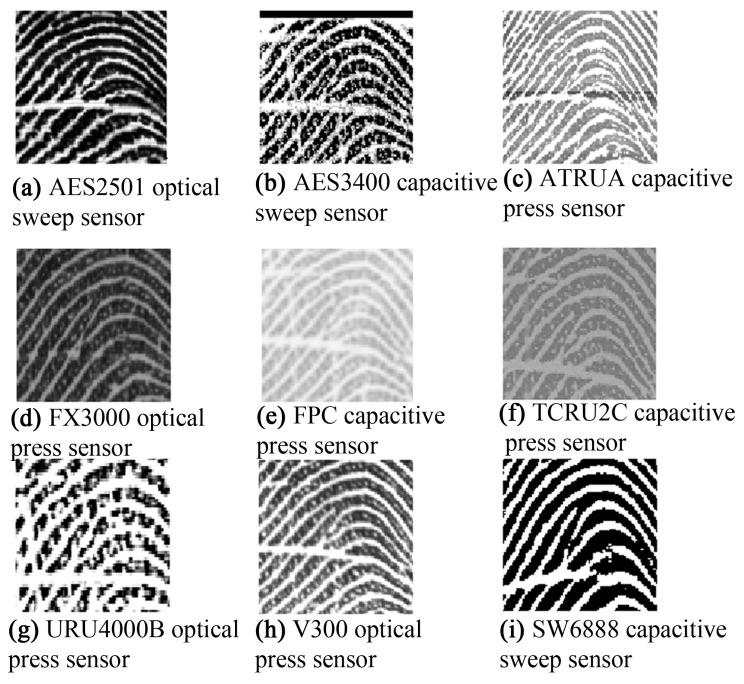
A zoomed-in view of fingerprints captured with different sensors.

**Figure 2:**
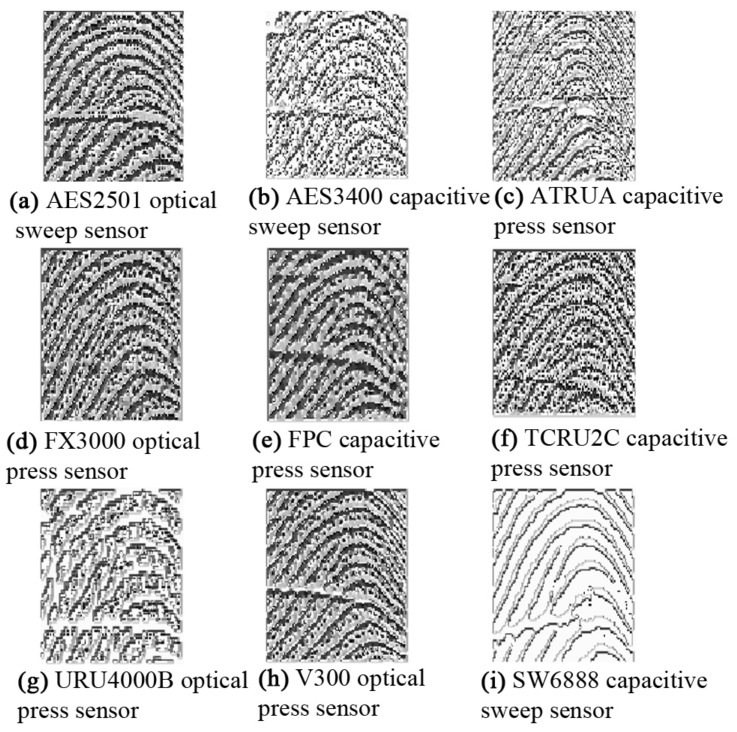
Corresponding local binary patterns (LBPs) for the fingerprints in Figure 1.

Texture features, such as local binary patterns (LBPs), histograms of oriented gradients (HoGs), and Gabor responses [6,7,8,9,10] are useful descriptors for a fingerprint-matching system; however, the texture of fingerprints varies because of the differences among sensors. Figure 1 shows zoomed-in views of some fingerprints of the same finger but captured with different sensors; the corresponding LBP images are shown in Figure 2. The LBP features differ from one another, showing large inter-class variations; there is concern regarding the ability of texture descriptors to discriminate the fingerprints captured with different sensors. The illustration reveals that texture is not a discriminative feature for cross-sensor matching. This variation complicates the search for a robust feature for fingerprint sensor interoperability.

**Figure 3:**
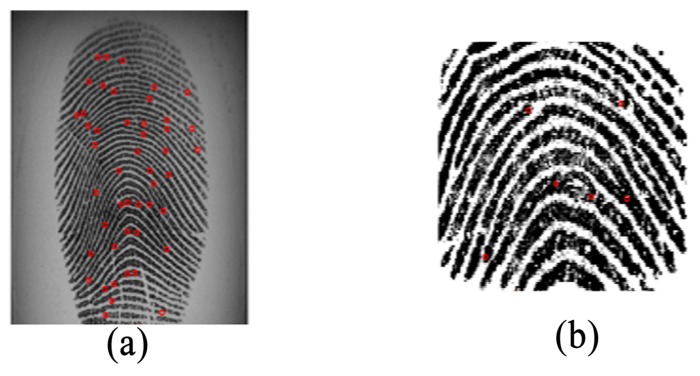
(**a**) Fingerprint from an FX3000 sensor and (**b**) the same fingerprint subject from an AES3400.

It can be observed in Figure 1 and Figure 2 that the ridge patterns are the same for all the views of fingerprints. These patterns are the most evident structural characteristics of a fingerprint and form strong features for use in discrimination [11]. Three levels are used to describe ridge details in fingerprints [1], namely the overall flow of the ridge pattern (Level 1), minutiae points (Level 2), and fine ridge details such as pores and edge contours (Level 3). The question arises whether these features are robust for fingerprint sensor interoperability. The global flow of ridge patterns remains the same in fingerprints acquired using different sensors, which indicates its robustness for discrimination. The minutiae points also remain the same, and these are strong discriminative features for recognition [12,13,14,15]. However, the sensor capturing area is a concern because a fingerprint captured by a sensor with a large capture area will always produce more minutiae than a sensor with a small capture area, as shown in Figure 3. This introduces a concern regarding fingerprint-matching systems based only on minutiae features. Missing minutiae because of a smaller capturing area may well affect discrimination. Fine ridge details, such as width, edge contour, and pores, are highly distinctive features [16,17,18,19]. However, as can be seen in Figure 2, these features cannot be reliably detected. For example, pores do not appear in Figure 2a,g,i and are hardly noticeable in the other fingerprints. Additionally, width and edge contours vary across the views in Figure 2. Thus, Level 3 features are not robust for the cross-matching problem.

The impact of sensor interoperability on fingerprint recognition has not been widely investigated. Exploring this problem will aid in understanding the effects of changing the sensors. There is a need to examine fingerprints captured via different sensors and their characteristics for the development of cross-sensor feature extraction and matching algorithms.

## 3. Datasets for the Fingerprint Sensor Interoperability Problem

Databases play a key role in evaluating the performance of a fingerprint matching system; however, few benchmark databases exist for the fingerprint interoperability problem. Such databases include the MCYT [20], GUC100 [21], ATVS-FFp [22], FingerPass [23], and MOLF [24] databases. These databases vary in terms of sensor technology, resolution, image size, capture method, and number of fingerprints used. The MCYT database includes only two different sensors with the same acquired resolution and capture methods. The ATVS-FFp database acquires its images using three different sensors; however, the total number of fingerprints used is the lowest of all the listed databases. Three different sensors were used to acquire the MOLF database data with the same sensor technology type and capture method. The fingerprints from the GUC100 database were captured with six different scanners and a variety of sensor technology types with the same resolution and capture method. The GUC100 is a semipublic database that requires researchers to either conduct testing at the premises of Gjøvik University College (Norway) or to submit algorithms in a compiled form to be run by researchers in Gjøvik. The FingerPass database includes sensors with two technology types and two interaction types, both of which differ in terms of resolution and image size. To address the challenges involved in fingerprint sensor interoperability, it is important to use a database with many variations in terms of the number of sensors used and their characteristics. None of the available databases include fingerprints from a variety of sensors of different technology and interaction types. There is a need to develop new databases that represent the various technology types and interaction types.

Among available public domain databases, FingerPass is the only large database that contains the maximum variety of cross-device fingerprints with different variations, which makes it a challenging database for a fingerprint recognition system. Table 1 provides a summary of the FingerPass database. It consists of nine datasets captured with different sensor types and interaction types.

## 4. An Overview of the State-Of-The-Art Methods

Most existing methods in the literature are designed for use with a specific technology type; consequently, their performance deteriorates when both gallery and probe fingerprints originate from different sensors. Recent work has shown the impact of diverse fingerprint sensor devices on the match error rate (EER) of fingerprint systems. Jain and Ross [25] investigated the problem of sensor interoperability by the collecting fingerprints of 160 individuals with optical and capacitive sensors. They studied the performance of some matching systems to match fingerprints that were obtained with different sensors, a significant drop was found in their performance. The inter-device EER increased to 23.13% when fingerprints collected from an optical sensor were matched with those by a capacitive sensor. Modi et al. [2] studied the impact of fingerprint sensor interoperability on the performance (in terms of false non-match rates) of a minutiae-based matcher. They formulated a statistical analysis framework for examining the similarities between minutiae count, fingerprint quality, and the performance on native and interoperable datasets. Lugini et al. [3] analyzed the sensor interoperability problem from a statistical perspective to measure the change in match scores when the sensors used for enrollment and verification differed. This study was performed on a private database, which was collected using four different optical sensors in addition to the scanned versions of ink-based fingerprints. Mason et al. [4] studied the effects of interoperability on different matchers using the same dataset as was adopted in [3]. These studies show that there is a significant impact of fingerprint sensor interoperability on matching performance of existing automatic fingerprint recognition systems; the performance drops significantly when different sensors are used for enrollment and query.

Few studies have focused on minimizing the effects of sensor interoperability. To address the effects of low interoperability between optical sensors, Marasco et al. [26] proposed an approach that employs various types of features and a classifier, which was developed for both cross- and intra-device matching. The adopted features were based on image quality, fingerprint-intensity-based characteristics, and minutia counts. Experiments were performed on a private database. The results showed that this approach improves cross-device matching in terms of false non-match rates. It was observed that it is only intensity-based features that vary by the type of sensor used for capturing fingerprints. In [5], a nonlinear calibration method was proposed to tackle the sensor interoperability problem using a thin-plate spline (TPS) model. This technique produces an average deformation model that defines the spatial relationship between two sensors. This method is not completely automated; the parameters depend on manually selected control points.

Some works have investigated the effect of scale on cross-sensor matching and have made some improvements. Ren et al. [27] proposed a scheme based on the average inter-ridge distance to compute the scale required to generate zoomed-in views of two fingerprints to be matched. The experiments were performed on FVC2002, which are not cross-sensor databases. Zang et al. [28] proposed a method for estimating the optimal scale between two fingerprints. In this method, the global scale is first computed coarsely using the ridge distance map, and a histogram of the local refined scale is then determined among all matchable minutiae pairs. The method was evaluated using four datasets from the FingerPass database. In [29], the Minutia Cylinder-Code was modified by introducing scale information. These studies indicate that the incorporation of scale information enhance the cross-device fingerprint matching performance.

As obvious from the above discussion that few studies have investigated the fingerprint sensor interoperability problem, their contributions toward solving this problem are marginal. Consequently, it remains a challenge. The main problems, such as fingerprint texture variation and the distortions in fingerprints that arise when using different sensors, have not yet been addressed properly.

## 5. Fingerprint Enhancement Methods

Fingerprint enhancement methods play an important role in improving image quality by enhancing ridge structures prior to feature extraction [30]. The role of enhancement methods becomes crucial when addressing the cross-sensor matching problem because fingerprints captured with different sensors include different types of noise and micro-texture patterns. To assess the potential of existing fingerprint enhancement methods on the fingerprint sensor interoperability problem, we analyzed the impact of two state-of-the-art methods: (i) HONG, the method employed by Hong et al. [31], in which the fingerprint is enhanced by applying a bank of Gabor filters that are tuned to the orientation of the local ridges, and (ii) CHIK, the method employed by Chikkerur et al. [32], in which fingerprint enhancement is performed using the short-time Fourier transform (STFT). In CHIK, the fingerprint is first divided into small overlapping windows, and the STFT is applied on each window. The ridge frequency, ridge orientation, and block energy are estimated based on the Fourier spectrum. Contextual filtering is then applied to enhance the fingerprints.

**Figure 4:**
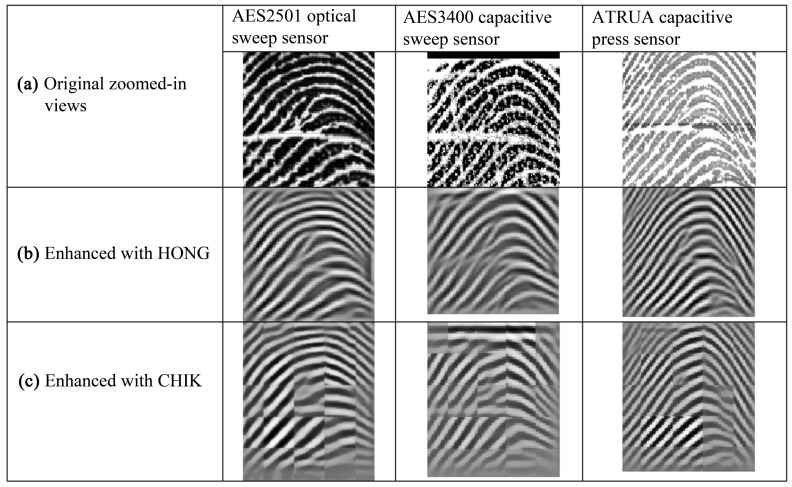
(**a**) Fingerprints from the same finger captured with three different sensors, (**b**) the corresponding fingerprints enhanced using the Hong et al. method, and (**c**) the corresponding fingerprints enhanced using the Chikkerur et al. method.

Figure 4 shows zoomed-in views of three fingerprints captured with three different sensors and their enhanced versions. The smoothed ridges of the enhanced fingerprints processed by HONG are better than those by CHIK; however, a close look reveals that both enhancement methods fail to preserve the original ridge patterns of the fingerprints and produce extraneous minutiae points and ridges. As a result, these two methods cannot be considered robust to address the challenges involved in sensor interoperability.

As a proof of concept, we performed three experiments using the VeriFinger and MCC algorithms (the detailed descriptions can be found in Section 7.1) to test the effectiveness of enhancement; Table 2 shows the results of the experiments. The EERs are lower after enhancement and HONG outperforms CHIK. Although, the results are better after enhancement, the improvement is not significant, it is likely due to the reason that both enhancement methods do not preserve precisely the ridge patterns and minutiae, which do not change no matter which sensor is used and form the discriminative content in case of cross-sensor matching. As different sensors result in different types of noises and texture microstructures because of their technology and capture types, these methods fail to produce consistent results under such variations. It necessitates the development of fingerprint enhancement methods which are invariant to technology and capture types of sensors and produce consistent enhancement results under various types of noise and variations of texture micro-structures.

## 6. Fingerprint Scaling and Its Impact on Cross-Sensor Matching

**Figure 5:**
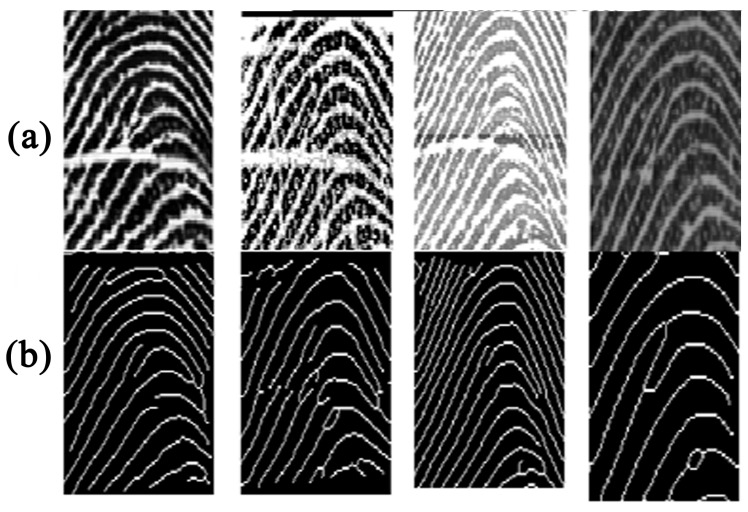
(**a**) Magnified views of fingerprints from the same finger captured with different sensors; (**b**) the corresponding thinned images.

A fingerprint is composed of connected ridges. The inter-ridge distance is an important fingerprint feature [1,33], but it is an issue with regard to fingerprint sensor interoperability [12,13,14,15]. It is measured as the average distance between two neighboring ridges [34]. Figure 5 shows four fingerprints from the FingerPass database [35] that were acquired from the same subject using four different sensors; the corresponding thinned fingerprints are given alongside for comparison. The spacing between successive ridges in the thinned fingerprints varies among the impressions captured with different sensors; i.e. the scales are different, which causes the failure of a genuine fingerprint match. This problem rarely exists in regular matching scenarios where fingerprints are obtained from the same sensor.

**Figure 6:**
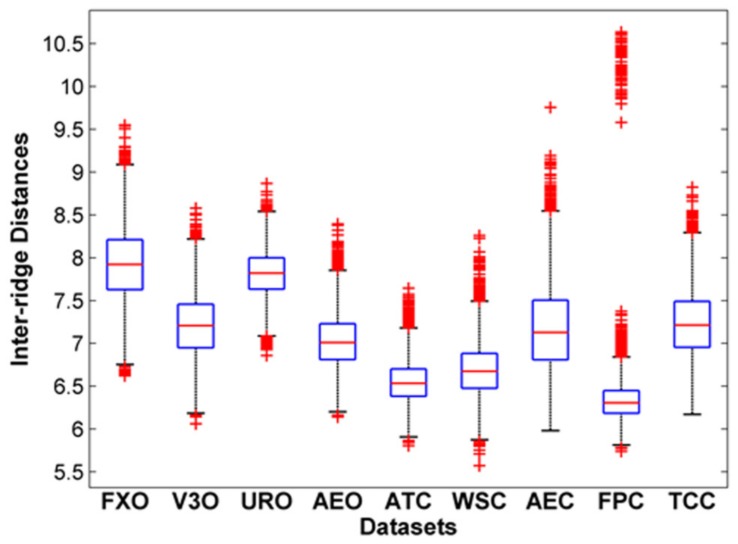
Box plot of inter-ridge distances.

Figure 6 shows the box plots of inter-ridge spacing for each dataset of the FingerPass database. ATC and FPC contain fingerprints with shorter inter-ridge distances compared to other datasets.

It has been shown that the scale of a fingerprint has impacts on cross-sensor matching [28]. To explore the effect of scaling, we performed three experiments using the VeriFinger and MCC algorithms. We first computed the average inter-ridge distances of two fingerprints being compared and computed the required scaling of the probe fingerprints as the quotient of the inter-ridge distances of the compared fingerprints. Table 3 shows the results of these experiments; the EERs are reduced to a great extent after scaling, which demonstrates the importance of scaling. Although some studies suggested adding a scaling step to fingerprint matching systems, time complexity remains an issue for the existing scaling-based method, and there is a need to extract features that are robust to scale variations.

## 7. Performance Analysis of the State-Of-The-Art Matching Methods

In this section, we present a detailed performance analysis of the state-of-the-art fingerprint matching methods for cross-sensor matching. First, an overview of the methods is given, and the evaluation protocol is then described.

### 7.1. Fingerprint Matching Methods

Although few studies have addressed the fingerprint sensor interoperability problem while modifying native matchers, the contributions are marginal and the main focus has remained on adjusting distortions [5,29]. The main issues that arise when using different sensors have not been addressed.

Minutiae-based methods are widely used for fingerprint matching; most of them employ minutiae descriptors. These methods are dominant because the information related to minutiae is highly discriminative and other features can be easily incorporated into minutiae descriptors. Our analysis of fingerprint structures in Section 2 reveals that minutiae are robust fingerprint features. Thus, this study focused on evaluating three state-of-the-art minutiae-based matchers, which are considered the baseline for comparing various research works for regular matching and cross-matching [29,35].

*MCC* [36] is a state-of-the-art minutiae-based matching algorithm. It is based on a 3D data structure called a cylinder, which is constructed from the distances of minutia points and orientations. The cylinder structure is a translation and rotation invariant with a fixed length of coding. *Bozoroth3* [37] is a minutiae-based matching system developed by the National Institute of Standards and Technology (NIST). It uses only the locations and orientations of minutiae to perform matching. It is also a translation and rotation invariant algorithm. *VeriFinger* [38] is a well-known commercial-matching algorithm developed by Neurotechnology. It is also based on minutiae and uses minutiae along with other properties.

For evaluation, we used VeriFinger Extended SDK 9.0, MCC SDK Version 2.0, and NBIS SDK Version 5.0 for Bozoroth3. It should be noted that both VeriFinger and NBIS use their own minutiae extraction algorithms, whereas MCC does not have a minutiae extraction algorithm. For MCC, we employed the minutiae extraction algorithm proposed in [31].

### 7.2. Evaluation Protocol

Our focus is on fingerprint verification or authentication in which the identity claim is verified. To evaluate the matching performance, we employed well-known metrics. The equal error rate (EER) is the basic metric for assessing performance. The EER is the operating point at which the FMR (false match rate) and the FNMR are equal. The FMR is the rate at which the matching method falsely considers two different fingerprints to be from the same person. The FNMR is the rate at which the matching method considers the fingerprints of the same person to come from different persons.

For evaluating the methods to assess the fingerprint sensor interoperability problem, we consider two matching scenarios: (i) *Regular Matching*, a comparison of two fingerprints acquired with the same sensor (also called native device or intra-device matching), in which case EER is referred to as native EER, and (ii) *Cross Matching*, a comparison of two fingerprints captured with different sensors (also known as cross-device or inter-device matching), in which case EER is termed as interoperable (cross) EER.

### 7.3. EER Analysis and Discussion

Table 4, Table 5 and Table 6 show the EER values produced by VeriFinger, MCC, and Bozoroth3, respectively. The results of VeriFinger are given in Table 4, which shows that all native EERs are much smaller than the interoperable EERs, except for AEC and FPC, which are 12.83% and 5.20%, respectively. For most of the cross-sensor matching cases, the cross EERs are high. In the case of cross-sensor matching when both probe and template sensors (FXO, V3O, and AEO) are of the optical type, the cross EERs are small, except for URO, which is also an optical sensor; regardless of whether or not URO is used as probe or template, cross EER is high, above 20%. Cross EER is high when both probe and template sensors are of capacitive type (ATC, SWC, AEC, FPC, and TCC). When optical sensors are used for the probe, and capacitive sensors are used for the template (or vice versa), the cross EERs are high except for TCC, which results in small EERs, regardless of whether they are employed as a probe or a template, except for URO. In a few cross-sensor cases, such as FXO and V3O, FXO and TCC, V3O and TCC, and V3O and AEO (where either can play the role of probe while the other acts as a template), the cross EERs are less than 1, whereas in most of the other cases it is much higher than 1. This indicates that in general the VeriFinger is not able to tackle the cross-sensor matching problem.

The MCC system results are shown in Table 5, which shows that the native EERs for optical sensors are much smaller than those for capacitive sensors. For most of the cross-sensor matching cases, the cross EERs are very high. In case of cross-sensor matching when both probe and template sensors (FXO, V3O, and AEO) are of optical type, cross EERs are relatively small, except URO, which is also an optical sensor. Regardless of whether or not URO is used as probe or template, cross EER is high, above 23%. Cross EERs are very high when both probe and template sensors are of capacitive type (ATC, SWC, AEC, FPC, and TCC). When optical sensors are used as probe, and capacitive sensors are used as template (or vice versa), the cross EERs are very high except for TCC, which results in relatively small EERs, regardless of whether they are employed as probe or template other than URO. In almost all cross-sensor matching cases, cross EERs are much higher. This indicates that in general the MCC performs the least effectively for cross-sensor matching problems.

The EER results for the Bozoroth3 method are presented in Table 6, which shows that all the native EERs (except for URO) and all the interoperable EERs are very high. This method performs poorly not only for cross-sensor matching but also for native matching, where probe and template fingerprints are captured from the same sensor.

From the above results and discussion, we can see that, although the VeriFinger outperforms the MCC and Bozoroth3 methods, it also produces poor results for cross-sensor matching. For further comparison, Table 7 summarizes the median and the mean EERs of cross-matching and regular matching for the three methods; note that the mean and median cross EERs were calculated when the listed dataset was used as a gallery set and the rest of the datasets were used as probe sets. This table also declares VeriFinger the winner, but note that it is not able to produce good cross-sensor matching results; the minimum median cross EER is 2.9 (when FXO is used as a gallery set) and the minimum mean cross EER is 8.6 (when V3O is used as a gallery set).

Table 7 indicates that VeriFinger has very high median and mean cross EERs on ATC, FPC, and URO datasets. ATC was collected with a capacitive sweep sensor, whereas FPC was obtained with a capacitive press sensor; these are the two databases with the lowest image resolution. URO was collected with an optical sensor of a press-interaction type. In contrast, the lowest median and mean interoperable EERs are obtained on V3O and FXO, which were captured using optical press sensors. Although the resolution of fingerprints affects the performance of VeriFinger, its matching results are not good even for high-resolution fingerprints. FXO, V3O, URO, and AEO also contain high-resolution fingerprints.

For MCC, the lowest median cross EER and the mean cross EER are 6.72% and 16.63%, respectively, which are much higher than those for VeriFinger; the highest cross EERs are on FPC, AEC, and ATC datasets, which were collected with capacitive sensors. In contrast, the lowest mean interoperable EER is obtained by V3O and FXO, which were captured using optical press sensors.

For Bozoroth3, the lowest median cross EER and the mean cross EER, were 42.52% on V3O and 37.91% on TCC, respectively, and these values are much higher than those for both VeriFinger and MCC. The highest cross EERs are on FXO, ATC, and FPC datasets; the ATC and FPC datasets were collected from capacitive sensors, whereas the FXO dataset was collected from an optical press sensor. Overall, the cross EERs of this method are much higher than those of VeriFinger and MCC. The three methods result in higher cross EERs on ATC and FPC, which is likely because of the resolution of the fingerprints.

**Figure 7:**
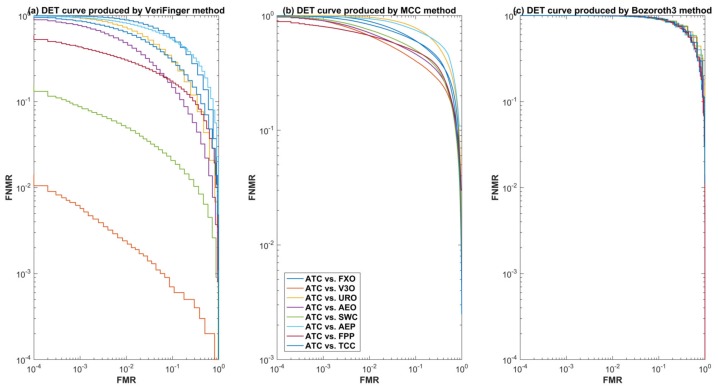
Detection error tradeoff (DET) curves of the three methods when ATC is used as a gallery set.

**Figure 8:**
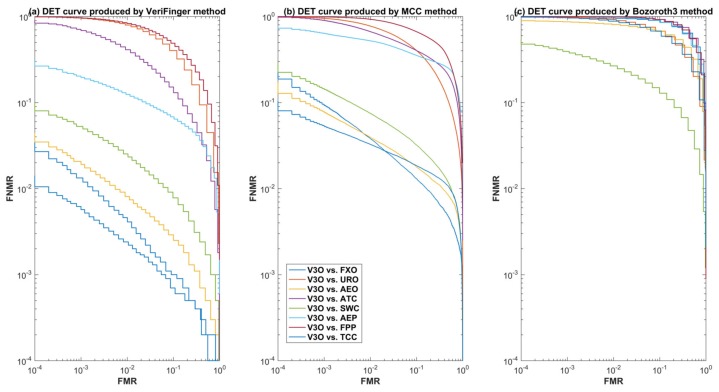
DET curves of the three methods when V3O is used as a gallery dataset.

For further insight into the performance of the three methods for cross-sensor matching, we selected one optical sensor (V3O) and one capacitive sensor (ATC) and plotted detection error tradeoff (DET) curves. Figure 7 and Figure 8 show the DET curves of the three methods for cross-device matching scenarios when ATC and V3O are used as a gallery and the rest of the datasets are used for the probe. These curves further validate that VeriFinger outperforms MCC and Bozoroth3 for cross-sensor matching, but its performance is also low.

### 7.4. Performance Analysis of Sensor Technology and Interaction Types

**Figure 9:**
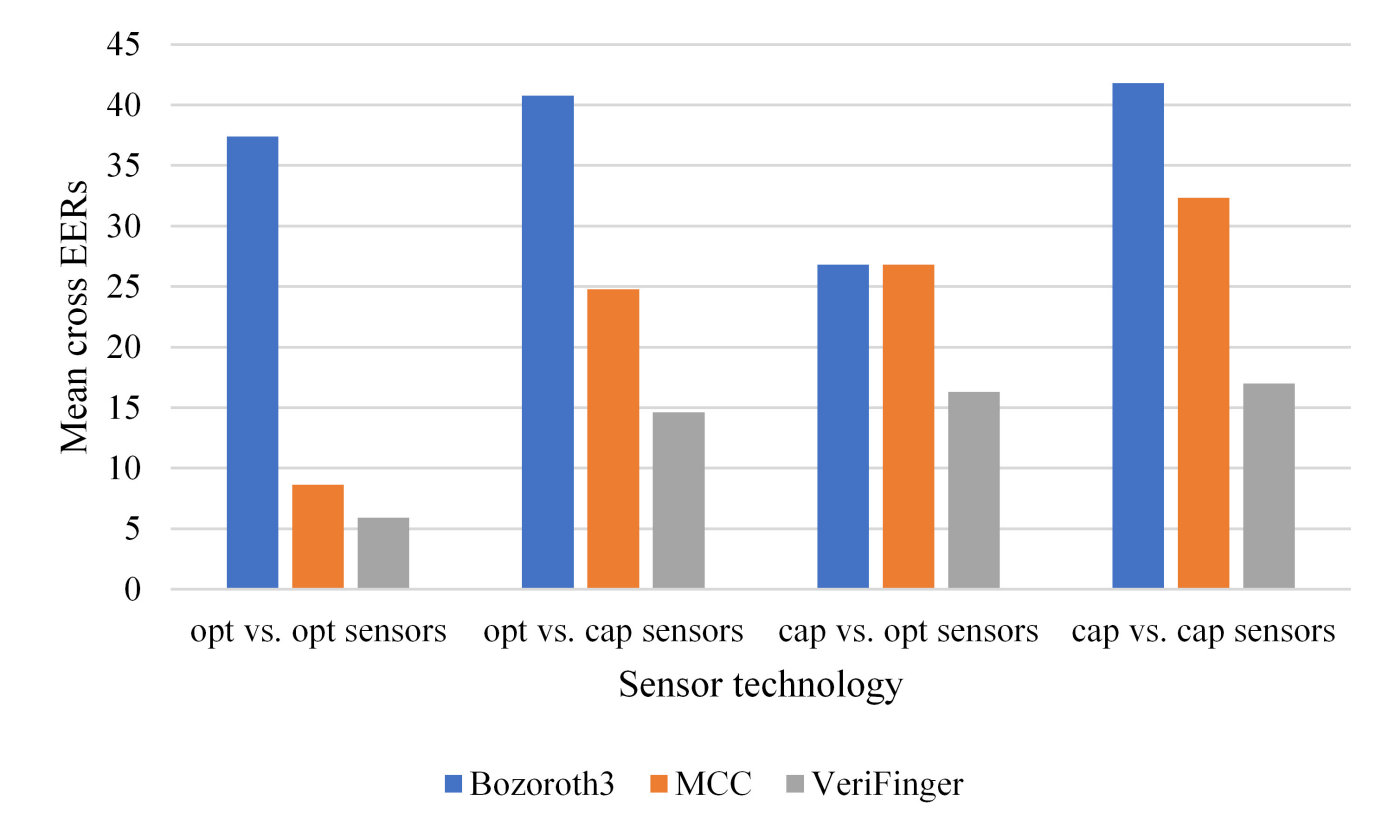
Mean cross EERs of the three methods for the sensor technology types; opt and cap indicate optical and capacitive sensors, respectively.

Further, we examine the cross-sensor matching performance of the three methods based on the technology types of the sensors. In this case, the datasets can be categorized into two groups: optical and capacitive. There are four cross-sensor matching scenarios: (i) optical vs. optical, where both the probe and the gallery are captured with optical sensors but of different types, (ii) capacitive vs. capacitive, where both the probe and the gallery are captured with capacitive sensors but of different types, (iii) optical vs. capacitive, where the the probe is captured with an optical sensor and the gallery is captured with a capacitive sensor, and (iv) the reverse of Scenario (iii). The optical group contains FXO through AEO, whereas the capacitive group includes ATC through TCC. Figure 9 shows the mean cross EERs of the three methods for the four scenarios. For each scenario, the mean cross EER was calculated considering all corresponding cross-matching cases as given in Table 2, Table 3 and Table 4 for each method. Figure 9 indicates that the lowest cross EER is obtained for the optical vs. optical scenario and that the capacitive vs. capacitive scenario results in the highest mean cross EER. Although the best performance for each group is obtained by VeriFinger, it also results in high mean cross EERs.

**Figure 10:**
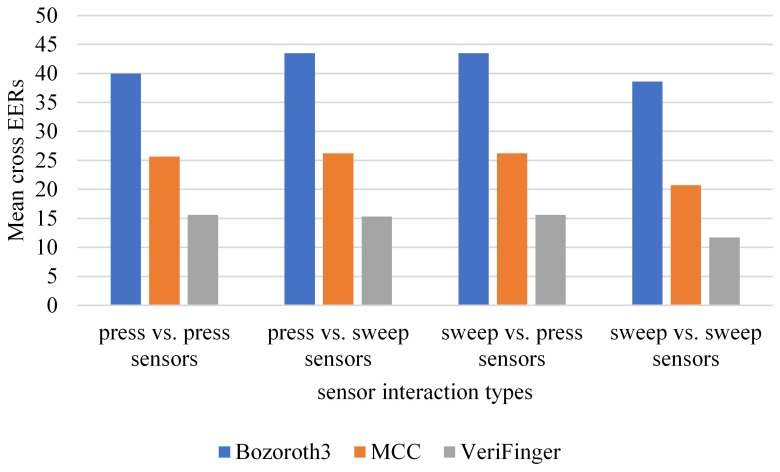
Mean cross EERs of the three methods for each sensor interaction type.

To examine the impact of sensor interaction type on cross-sensor matching, the datasets are grouped into two categories: press and sweep. There are four cross-sensor matching scenarios: (i) sweep vs. sweep, where both probe and gallery sets are captured with different sensors of the sweep interaction type, (ii) press vs. press, where both probe and gallery sets are captured with different sensors of the press interaction type, (iii) sweep vs. press, where the probe set is captured with a sensor of the sweep interaction type and the gallery set is captured with a sensor of the press interaction type, and (iv) the reverse of Scenario (iii). The sweep group contains AEO, ATC, and SWC, whereas the press group includes all other datasets. Figure 10 shows the mean cross EERs of the three methods for each scenario. The results show that the lowest mean cross EER is obtained with VeriFinger for the sweep vs. sweep scenario; the mean cross EERs for the other three categories are the same. In terms of the comparative performance of the methods, a trend similar to that in the case of technology types can be observed here; among the three methods, VeriFinger results in the lowest mean cross EERs, but its performance is also far from being optimal.

**Figure 11:**
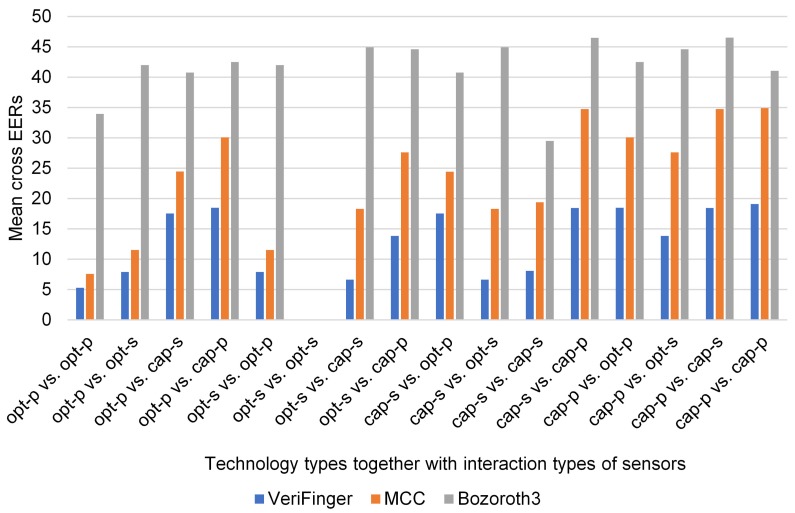
EERs of the three methods for technology types together with interaction types of sensors.

To examine the impact of technology types together with interaction types of sensors on cross-sensor matching performance, the mean cross EERs of the three methods are shown in Figure 11 for different cross-sensor matching scenarios. The datasets are categorized into four groups: optical-press (FXO, V3O, and URO), optical-sweep (AEO), capacitive-sweep (ATC and SWC), and capacitive-press (AEC, FPC, and TCC). It should be noted that there is only one dataset (AEO) in the category optical-sweep. There are 16 cross-sensor matching scenarios such as optical-press vs. optical-press, optical-sweep vs. optical-sweep, and optical-press vs. optical-sweep as shown in Figure 11. For each scenario, the mean cross EER was calculated from Table 4, Table 5 and Table 6 considering the corresponding cases, e.g., the mean cross EER for the scenario optical-press vs. optical-press was calculated by taking into account all cross-sensor matching cases where different optical-press sensors were used for the probe and gallery.

Figure 11 shows that among all scenarios (excluding optical-sweep vs. optical-sweep) the lowest mean cross EERs of all the three methods are for the optical-press vs. optical-press scenario; the mean cross EER of VeriFinger is the lowest among the three methods. Note that optical-sweep vs. optical-sweep is a native matching scenario because there is only one dataset for this scenario. For all other scenarios, the mean cross EERs are very high. Though VeriFinger has the lowest mean cross ERR (5.27%) (for the optical-press vs. optical-press scenario) among the three methods, this result is far from being optimal.

The performance of the three state-of-the-art methods was examined for regular matching, cross-sensor matching, and cross-sensor matching, keeping in view technology types, interaction types, and technology types combined with interaction types. This analysis shows that, although VeriFinger provides improved cross-sensor matching results in comparison with MCC and Bozoroth3, its performance is also not optimal for cross-sensor matching. This leads to the conclusion that there is a need to develop new algorithms for cross-sensor matching problems.

## 8. Conclusions and Future Work

We performed a large-scale analysis of the fingerprint sensor interoperability problem. We provide insight into the real issues involved in the fingerprint sensor interoperability problem. Low-level structural inconsistencies and distortions occur in fingerprints that are captured with different sensors. It is shown here that enhancement algorithms have a significant impact on cross-sensor matching. We also analyzed the cross-matching performance of three state-of-the-art methods on a public multi-sensor database.

Several variations are introduced in fingerprints captured with different sensors due to differences among sensors in terms of capturing technology, interaction type, sensor resolution, and scanning area. These variations appear as inconsistencies in fingerprint texture and fine ridge details such as width, pores, and inter-ridge spacing, making it difficult to extract discriminative descriptions of fingerprints, which are captured with different sensors. However, ridge patterns and minutiae points are invariant across different sensors and form the discriminative description of fingerprints for cross-sensor matching. The challenge is to enhance the ridge patterns and minutiae while suppressing small-scale inconsistencies such as micro-texture patterns and fine ridge details such as width and pores. To evaluate the potential of existing enhancement methods to meet this challenge, we analyzed the performance of two state-of-the-art enhancement algorithms. We found that, although an enhancement method can overcome this issue and improve cross-matching performance, existing methods designed for regular matching are not robust enough for cross-sensor matching. Therefore, there is a need to design interoperable enhancement algorithms that preserve ridge patterns and suppress inconsistent small details.

The performance analysis of three state-of-the-art methods shows that, when the same sensor is used for enrollment and verification, the native EER is generally very low, particularly for VeriFinger. Performance is significantly reduced, even for the best performing method VeriFinger, when different sensors are used for enrollment and verification. This highlights the research challenge of cross-device matching. VeriFinger was shown to be the best of the three matching methods; it yielded lower EER values than MCC and Bozoroth3. VeriFinger also uses minutiae along with several other properties such as ridge count, which highlights that fusing other features with minutiae can have a positive impact on reducing the interoperability problem.

An analysis of cross-sensor matching based on the technology types of sensors shows that optical sensors result in relatively low mean cross EERs. By contrast, the capacitive sensors yielded the worst mean cross EERs for all three matching methods. Therefore, the best choice for cross-sensor matching is to use optical sensors for enrollment and verification.

An analysis of cross-sensor matching based on the technology types and interaction types of sensors revealed that optical sensors with the press interaction type resulted in the lowest mean cross EERs for all three methods; by contrast, capacitive sensors with the press interaction type yielded the worst mean cross EERs. Furthermore, capacitive sweep vs. capacitive press (and vice versa) resulted in high mean cross EERs. Therefore, the level of interoperability between optical-press sensors is better than that between capacitive sensors, be they press or sweep.

In addition to the technology type and interaction type of sensors, the size and resolution of fingerprints generate an important effect on error rates. The impact of different transformation models, such as scaling and resolution compensation models, must be investigated on cross-sensor matching. The effect of technology type and interaction type of sensors on image quality should also be examined.

Most systems use only a single impression of the fingerprint to extract features. However, the effect of using multiple impressions of fingerprints to capture more features should be investigated for better results.

Our study of the fingerprint sensor interoperability problem shows that fingerprints captured with sensors of different technology types and interaction types involve different types of distortions and small-scale inconsistencies such as micro-texture patterns and pores. Moreover, the fingerprints differ in scale and resolution. The main structure that is invariant among fingerprints captured with different sensors consists of ridge patterns, ridge orientations, and minutiae. In view of this, future studies should develop fingerprint enhancement algorithms for cross-sensor matching that can enhance ridge patterns and minutiae and suppress inconsistencies such as micro-texture patterns. Fingerprints captured with different sensors vary in scale and resolution; cross-sensor matching requires the development of new extraction techniques that are robust against variations of orientations, scale, and resolution.

## Figures and Tables

**Table 1:** Summary of the FingerPass database.

Sub-Dataset	Sensor	Technology	Capture Method	Resolution (dpi)	Image Size (Pixels)
FXO	Biometrika FX3000	Optical	Press	569	400×560
V3O	CrosssMatch Verifier 300	Optical	Press	500	640×480
URO	Digital Persona URU4000B	Optical	Press	700	500×550
AEO	Authentec AES2501	Optical	Sweep	500	Unfixed
ATC	ATRUA	Capacitive	Sweep	250	124×400
SWC	Aymware SW6888	Capacitive	Sweep	500	288×384
AEC	Authentec AES3400	Capacitive	Press	500	144×144
FPC	FPC1011C	Capacitive	Press	363	152×200
TCC	UPEK TCRU2C	Capacitive	Press	500	208×288

**Table 2:** Match error rate (EER) of the VeriFinger and Minutiae Cylinder-Code (MCC) algorithms before and after enhancement.

Gallery	Probe	VeriFinger			MCC		
		Before Enhancement	HONG	CHIK	Before Enhancement	HONG	CHIK
AEO	AEC	10.63	6.959	8.306	34.71	29.0572	33.9610
AEO	FPC	28.98	21.007	27.422	41.25	35.4789	40.1459
FPC	AEC	38.03	29.0856	34.8476	47.53	39.1429	45.940

**Table 3:** EERs of the VeriFinger and MCC algorithms before and after enhancement.

Gallery Dataset	Probe Dataset	VeriFinger		MCC	
		Before Scale	After Scale	Before Scale	After Scale
AEO	AEC	10.63	3.92	34.71	18.30
AEO	FPC	28.98	17.77	41.25	30.28
FPC	AEC	38.03	23.15	47.53	33.56

**Table 4:** EER (%) produced by the VeriFinger system.

(Template/Probe)	FXO	V3O	URO	AEO	ATC	SWC	AEC	FPC	TCC
FXO	0.08	0.54	2.53	2.14	26.92	3.28	7.40	36.54	0.50
V3O	0.54	0.14	20.54	0.86	11.79	1.85	7.24	25.66	0.33
URO	2.52	20.49	0.01	20.62	41.47	19.75	27.81	43.30	17.44
AEO	2.14	0.86	20.65	0.04	12.08	1.16	10.63	28.98	1.85
ATC	26.89	11.79	41.47	12.09	0.67	15.56	30.32	14.14	19.06
SWC	3.27	1.85	19.77	1.16	15.57	0.45	12.83	31.01	3.28
AEC	7.39	7.25	27.82	10.62	30.33	12.81	12.87	38.07	8.04
FPC	36.56	25.66	43.35	28.99	14.13	30.99	38.03	5.20	30.62
TCC	0.50	0.33	17.45	1.86	19.06	3.28	8.03	30.66	0.17

**Table 5:** EER (%) produced by the MCC system.

(Template/Probe)	FXO	V3O	URO	AEO	ATC	SWC	AEC	FPC	TCC
FXO	0.38	2.53	7.15	4.69	37.16	6.29	31.80	43.98	4.88
V3O	2.53	0.90	23.53	2.85	28.49	4.51	28.88	39.67	2.60
URO	7.15	23.53	0.31	26.98	43.19	26.92	43.98	46.44	28.47
AEO	4.69	2.85	26.98	0.92	31.59	4.97	34.71	41.25	6.81
ATC	37.16	28.48	43.19	31.59	9.48	32.51	47.69	33.48	37.83
SWC	6.28	4.51	26.92	4.96	32.50	3.07	36.88	41.45	11.11
AEC	31.80	28.88	43.98	34.71	47.70	36.88	43.18	47.53	32.05
FPC	43.98	39.67	46.44	41.25	33.48	41.45	47.53	25.37	41.89
TCC	4.88	2.60	28.47	6.81	37.83	11.11	32.05	41.89	2.50

**Table 6:** EER (%) produced by the Bozoroth3 method.

(Template/Probe)	FXO	V3O	URO	AEO	ATC	SWC	AEC	FPC	TCC
FXO	38.51	47.98	49.72	49.03	49.64	49.00	48.68	50.44	49.33
V3O	47.97	2.65	34.16	38.03	49.44	11.37	47.01	50.03	33.50
URO	49.72	34.14	0.58	38.85	49.42	35.66	45.70	49.99	7.83
AEO	49.02	38.06	38.84	41.33	50.18	39.70	47.53	50.71	35.56
ATC	49.60	49.43	49.43	50.17	12.43	49.98	48.69	50.16	49.06
SWC	48.97	11.38	35.68	39.69	49.97	5.51	47.18	50.92	32.90
AEC	48.67	47.02	45.69	47.52	48.70	47.21	36.54	48.54	45.29
FPC	50.45	50.05	49.96	50.71	50.24	50.91	48.56	40.95	49.68
TCC	49.32	33.55	7.88	35.56	49.04	32.92	45.31	49.71	4.95

**Table 7:** Native EER, median, and mean cross-device EER values produced by the tested systems.

(Template/Probe)	VeriFinger			MCC			Bozoroth3		
	Native EER	Median cross-EER	Mean cross-EER	Native EER	Median cross-EER	Mean cross-EER	Native EER	Median cross-EER	Mean cross-EER
FXO	0.08	2.9	9.98	0.38	6.72	17.31	38.51	49.18	49.23
V3O	0.14	4.55	8.6	0.9	14.02	16.63	2.65	42.52	38.94
URO	0.01	20.55	24.17	0.31	27.72	30.83	0.58	42.28	38.91
AEO	0.04	6.39	9.79	0.92	16.89	19.23	41.33	43.62	43.7
ATC	0.67	17.31	21.41	9.48	35.32	36.49	12.43	49.51	49.56
SWC	0.45	8.05	11.09	3.07	19.02	20.58	5.51	43.44	39.59
AEC	12.87	11.72	17.79	43.18	35.79	37.94	36.54	47.36	47.33
FPC	5.2	30.8	31.04	25.37	41.67	41.96	40.95	50.15	50.07
TCC	0.17	5.66	10.15	2.5	19.79	20.71	4.95	40.44	37.91
